# In-Hospital Death Caused by Pancreatic Cancer in Spain: Application with a Bayesian Network

**Published:** 2011-06

**Authors:** A. Álvaro-Meca, R. Gil-Prieto, A. Gil de Miguel

**Affiliations:** *Department of Preventive Medicine and Public Health, University Rey Juan Carlos, Spain*

**Keywords:** bayesian networks, max-min hill-climb algorithm, pancreatic cancer, in-hospital death

## Abstract

Pancreatic cancer is one of the least common tumors (2.1%), but it remains one of the most lethal. This lethality is primarily due to late stage diagnosis in the vast majority of patients. Here we demonstrate, using a Bayesian network, that we can determine a posteriori, with a high probability of success, the probability of in-hospital death of pancreatic cancer in hospitals across Spain with information related to the type of admission, the type of procedure, the primary diagnosis or the Charlson co-morbidity index. The advantages of using a Bayesian network are that it allows us to examine multiple hypotheses and to measure the effect of the introduction of variables on our hypotheses. Being able to determine deceases in the probability of survival based on hospital admission data, such as the diagnosis resulting in the present admission or the presence of co-morbidities, could facilitate the detection of deficiencies in the patient treatment and improve hospital management. Moreover, the control of related co-morbidities may have an impact on the in-hospital deaths of these patients.

## INTRODUCTION

Pancreatic cancer is one of the least common tumors (2.1%), but it remains one of the most lethal. This lethality is primarily due to late stage diagnosis in the vast majority of patients. Recently, pancreatic cancer has become equally prevalent in men and women as a result of the increasing exposure of women to nicotine poisoning, one of the main risk factors. The study of factors that may determine the probability of in-hospital death caused by this kind of tumor is interesting, and improvement in mortality rates will have significant social and economic consequences.

Cancer data processing is usually carried out using multi-state models (23) that allow prediction of the probability of future events for a particular patient at a certain point in time given the covariates and clinical history of that patient. Nonetheless, other models are applicable in some specific cases. For instance, Clements *et al*. (2005) (3) suggest using Generalized Additive Models to forecast lung cancer mortality rates and additionally assert that “Predictions of future cancer incidence and mortality rates, defined here as rate projections, are necessary for planning public health programs and clinical services, including clinical training (9)”. We propose an analysis using a Bayesian Network as it provides us not only with information about probabilistic dependences between variables but also the conditional independence of a variable or groups of variables from a given variable or variables. Each variable is independent from variables that are not descendent in the graph, given the state of their parents’ variables. The inclusion of the independence relationship in the graph structure makes the Bayesian Network a powerful tool to represent knowledge in a compact way. Furthermore, it provides us with flexible methods for reasoning based on the propagation of probabilities through the network, as expected following probability theory.

A Bayesian Network (13, 22) is a graphical model of probability relations, *P*, among a set of variables, *X*. Because information is accessed by a retrieval engine, Bayesian networks are an extremely useful tool for updating probabilities when new evidence becomes available. The primary aim of this study is to determine the probability of in-hospital death due to pancreatic cancer in Spain during the year 2007 by working within a graphical probabilistic model framework; to be exact, we employ a graphical casual probabilistic model defined using a Bayesian Network (2).

## MATERIALS AND METHODS

### Data Sources

Hospital discharge data related to pancreatic cancer were extracted from the Minimum Basic Data Set (MBDS), the hospitalization data collection system used across Spain. Until 2004, this database managed clinical codes from the Spanish version of the Ninth International Classification of Diseases (ICD-9MC). Since 2004, the Tenth International Classification of Diseases has been applied. It is estimated that the MBDS collects data from 97% of hospital discharges from public hospitals and 25% of those from private clinics (18, 24). Private hospital-related cases represent a small proportion of all hospital admissions because the vast majority of Spain’s population is covered by Public Health-Care Insurance (10).

For this study, all pancreatic cancer hospitalizations (ICD-9CM 157.0-157.9; as the primary diagnosis) occurring between January 1 and December 31, 2007 were included. Information obtained from the MBDS included sex, age, diagnosis, type of procedure, type of hospital, type of admission and outcome (survival to hospital discharge or death). Additionally, for each case we calculated the Charlson co-morbidity index (CCI), which has been proven to be an effective predictor of morbidity and mortality in patients with colorectal carcinoma (20) and of in-hospital death, as demonstrated by Librero *et al*. (1999) (17). The CCI weights utilized in this study are presented in Table 1. To carry out the CCI calculation, only secondary diagnoses were considered. The final rating for each record includes any additionally obtained information, where a single illness is counted in each of the groups.

**Table 1:** Charlson Comorbidity Index

Condition	Weight

Myocardial infarction (MI)	1
Congestive heart failure (CHF)	1
Peripheal vascular disease (PVD)	1
Cerebrovascular disease (CVD)	1
Dementia	1
Chronic obstructive pulmonary disease	1
Connective tissue disease	1
Ulcer disease	1
Mild liver disease	1
Diabetes	1
Hemiplejia	2
Moderate/severe rental disease	2
Diabetes with end-organ damage	2
Any Tumor	2
Leukemia	2
Lymphoma	2
Moderate/severe liver disease	3
Metastatic solid tumor	6
Aids	6

### Statistical analysis: Bayesian Networks

A Bayesian Network is described as a pair consisting of a qualitative portion and a quantitative portion, where the qualitative portion of the model is determined using a Directed Acyclic Graph (*DAG*). For a *DAG, G=(V, E)*, where *V* denotes a set of nodes (or variables) and *E* denotes a set of directed links (or edges) between pairs of the nodes. A joint probability distribution, *P*(*X_v_*), over the set of (typically discrete) variables, *X_v_*, indexed by *V*, can be factored as

(a) $$P\left(X_{V}\right)=\prod_{v\in V}P\left(X_{v}|X_{\mathrm{pa}\left(v\right)}\right)$$

where *X_pa(v)_* denotes the set of parent variables of variable *X_v_* for each node *_v e V_*. The factoring in Equation 1 expresses a set of independent assumptions that are represented by the *DAG* in terms of pairs of nodes that are not connected to one another by a direct link.

**Inference in Bayesian Networks.** An important component of a Bayesian Network is its inference engine. Introducing evidence (information) concerning one or more of the variables in the network allows us to update the a posteriori probabilities of all the nodes in the network. The propagation of evidence may be exact or approximate. While the exact inference of variables is known to be an NP-hard problem (5), approximate algorithms are also NP-hard (7). In our study, we utilize exact algorithms, as this method allows us to obtain the probabilities of nodes accurately and without error.

A number of propagation algorithms have been developed. Olmsted (1983) (19) and Shachter (1988) (27), who stand out in the field of the development of Discrete Bayesian Networks, developed an algorithm based on allowing *DAG* edges to become reversible, such that the answer to the probabilistic question can be read directly from the graph, allowing the probability associated with each reversible edge to be obtained prior to the application of the Bayes Theorem. Kim *et al*. (1983) (14) and Pearl (1986) (21) developed the “pass the message” diagram, which updates the probability distribution of each node of the Bayesian Network when there is evidence of one or more variables. Lauritzen (1988), Jensen *et al*. (1990a, b) (16, 11, 12) and Dawid (1992) (8) developed an algorithm based on transforming the initial *DAG* to represent the Bayesian Network as a tree where each of the nodes is made up of a subgroup of variables, *X*. This algorithm uses the diverse mathematical properties of the tree to carry out the process of evidence propagation. D’Ambrosio (1991) (6) subsequently developed an evidence propagation algorithm that simplifies a portion of the calculations of a previously specified algorithm. However, the algorithm described by Lauritzen (1988), Jensen *et al*. (1990a, b) (16, 11, 12) and Dawid (1992) (8) remains the most used in Discrete Bayesian Networks and is the one that we use in this study.

**Figure 1:**
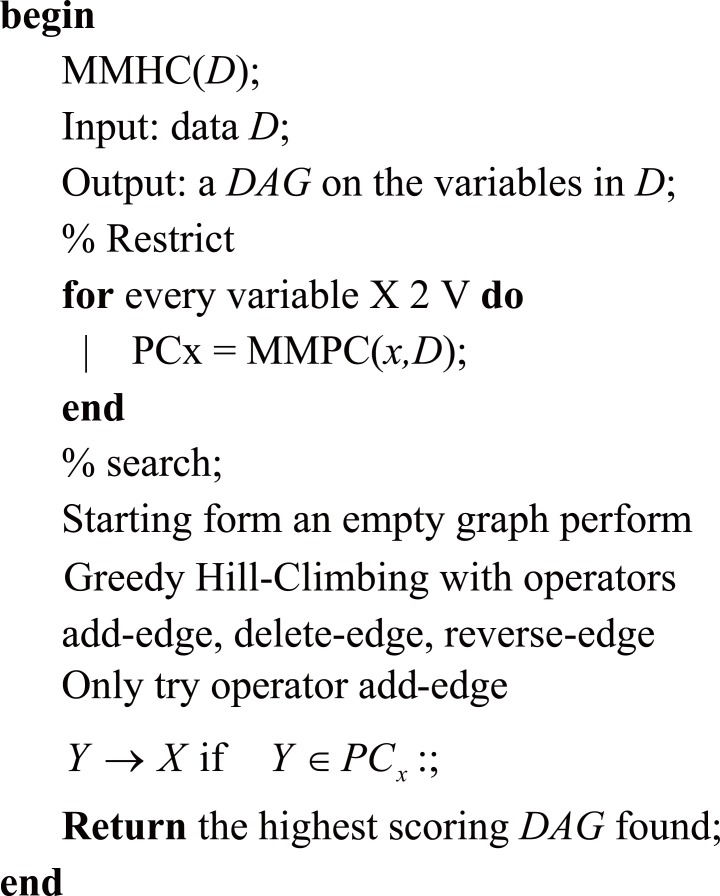
MMHC algorithm.

**Structure learning from data.** Structure learning from data is the task of including the structure, i.e., the graph, of a Bayesian Network from source data. Different classes of algorithms are available for determining the structure of a Bayesian Network (15). Here, we use the structure learning algorithm known as Max-Min Hill-Climbing (MMHC) that was developed by Brown *et al*. (2004) (1) and implement it in R with the *bnlearn* package developed by Scutari (2009) (26). The Max-Min Hill-Climbing algorithm combines local learning ideas and constraint-based and search-and-score techniques in a principled and effective way. It first reconstructs the skeleton of a Bayesian network and then performs a Bayesian-scoring greedy Hill-Climbing search to orient the edges (28). The algorithm MMHC is described in Figure 1.

For more details of this algorithm see Tsamardinos *et al*. (2006) (28).

## RESULTS

### Selection of the Network Structure

We selected 6,579 patients with a primary diagnosis of pancreatic cancer; 56% were men, and the average age at hospital discharge was 68.07 [CI95% (67.77, 68.38)]. The data are summarized in Table 2, and the variables considered for the Bayesian Network included the age of the patient, the Charlson co-morbidity index, the type of admission, the type of DRG or type of procedure carried out (medical or surgical), the result of the process (mortality), the hospital group and the primary diagnosis.

**Table 2:** Description of the patients

Caracteristic	N	%

Age		
<50	642	9.76
50-59	1030	15.66
60-69	1114	16.93
70-79	1669	25.37
≥80	2124	32.28
CCI		
No comorbidity	1608	24.44
1-6	3335	50.69
>6	1636	24.87
Type of admission		
Urgent	1900	28.88
Scheduled	4679	71.12
Type of procedure (DRG)		
Medical	4912	74.66
Surgical	1667	25.34
Outcome		
Alive	4891	74.34
Death	1688	25.66
Hospital Group		
Type 1	847	12.87
Type 2	1497	22.75
Type 3	1882	28.61
Type 4	2353	35.77
Main Diagnosis		
N. Malignant head of pancreas	2710	41.19
N. Malignant body and tail of the pancreas	793	12.05
N. Malignant other specified areas	715	10.87
N. Malignant other non-specified areas	2361	35.89

With regard to the determination of the structure of the network, we used the MMHC algorithm previously described in section **Structure learning from** data. This algorithm, proposed by Tsamardinos *et al*. (2006) (28), was selected after it proved to be superior to other candidate algorithms based on a number of metrics. However, to determine the structure of the data, we additionally applied alternate algorithms, including Hill-Climbing (HC) and Max-Min Parents and Children (MMPC). BIC was used as a score. The results obtained were similar to those obtained using the MMHC algorithm; however, the computational time was slightly shorter when the MMHC algorithm was used (data not shown).

**Figure 2:**
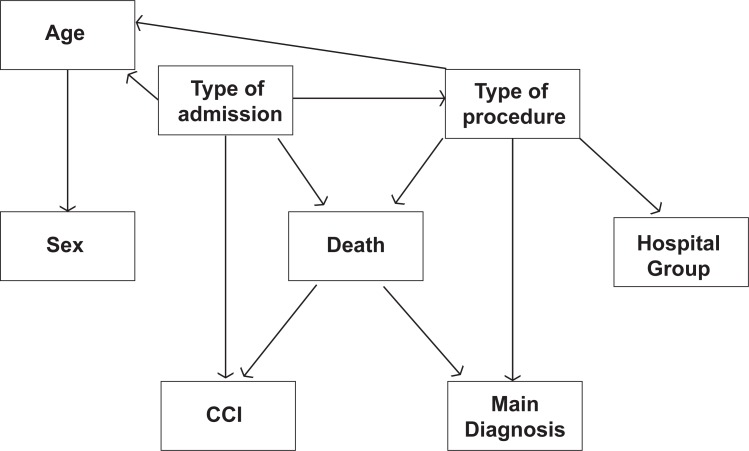
Bayesian Network.

The variables included were selected based on previous studies (25) and the knowledge of hospital discharge database experts. Using the MMHC algorithm, we obtained 1,000 bootstrap samples of the data and determined a network for each one. The optimal network was determined according to the most frequently recurring network and validation in consultation with experts. This optimal network is presented in Figure 2. We observed the occurrence of arrows indicating a reverse pathway from death → CCI and death → Main Diagnosis. Contrary to the suggestion that the network was invalid, the direction of the arrows was consistent with a database of hospital discharge information in which the last data point provided corresponded to the diagnosis (Main Diagnosis and CCI). Information provided prior to diagnosis, including age, gender, type of admission and type of procedure (medical or surgical), is also available. Following determination of the network structure, network parameters that will aid in the making of inferences must be defined. Parameter learning is a simple process when all the variables are fully observable. The most common method currently used is known as the Maximum Likelihood estimator, which estimates the desired probabilities from the frequency of training data values, which is analogous to Naive Bayes.

### Analysis of network performance

With regard to our network, small hospitals and group 1 or 2 hospitals are associated with a higher probability of death than group 3 or 4 hospitals. This is primarily due to the decreased likelihood that a surgical procedure will be carried out for this kind of tumor at a small hospital; admission to small hospitals has a palliative purpose rather than a curative one. Moreover, when surgical procedures are included as evidence in the network, there is a lower probability of death (0.147) than when they are excluded from the network (0.256).

As has already been demonstrated, patient age is associated with mortality, such that as age increases, the probability of in-hospital death increases. Thus, the probability of in-hospital death for patients less than 50 years of age is 0.214, for patients from 50 to 59 the probability of in-hospital death is 0.245 and for patients over 59 the probability is 0.248. For older patients the effect of age is larger, with the probability of in hospital death increasing to 0.260 and 0.284 for the 70-79 and more than 80 years old groups, respectively.

When it is included in the network, the presence of a primary diagnosis of “N. Malignant other non-specified areas”, code ICD 9-CM 157.9, results in an in-hospital death probability of 0.339, which is higher than that obtained when the location of the tumor is known, except when the tumor is located in the tail of the pancreas. This coincides with the natural history of the disease because tumors located in the tail of the pancreas typically appear in the advanced stages of hepatic metastases or peritoneal carcinomatosis. In the same way, tumors of an uncertain location (code ICD 9-CM 157.9) present a worse prognosis, perhaps related to more advanced clinical profiles, as has been previously indicated.

Consistent with the conclusions of Sendra *et al*. (2008) (25), when the Charlson co-morbidity index is included in the network it has a great impact in the a posteriori probability of in-hospital death, highlighting the importance of co-morbidity in determining the final outcome. CCI values above 6 increased the probability of death to 0.361, and the lack of co-morbidity reduced the probability of death from 0.256 to 0.163, indicating the importance of controlling co-morbidities in these patients.

## DISCUSSION

One of the great advantages of using a Bayesian Network is that it not only allows us to study several hypotheses simultaneously, it allows us to observe the effect of the introduction of information (evidence regarding a given variable) on our hypothesis. One of the main conclusions that we have drawn here is that when information about the type of admission, type of DRG, primary diagnosis or CCI is provided, we can determine a posteriori, with a high probability of success, the probability of in-hospital death from pancreas cancer across hospitals in Spain.

As far as we know, no one has developed a model of pancreatic cancer in-hospital deaths in Spain using a Bayesian Network. Sendra *et al*. (2008) (25), using the MBDS from 2004, performed a logistic regression and obtained results similar to those obtained here for the year 2007. This provides us with insight into the slow improvement in in-hospital deaths with this kind of tumor. Similarly, we also identified shortcomings in using an administrative database. The primary shortcomings are related to the limited and non-specific nature of the clinical information included in the database, particularly with regard to the treatment provided to patients, patient records and risk factors. Additionally, we note the inability to differentiate between a co-morbidity and a complication.

We plan to propose the development of a dynamic Bayesian Network, with the aim of detecting trends and executing projections of in-hospital deaths due to pancreas cancer in the future, as well as extending the network to other kinds of tumors.

The ability to determine the probability of death given previous information about admission, such as the diagnosis that resulted in the admission and the presence of co-morbidities, could have great advantages for hospitality management, not only because deficiencies in the treatment of these patients could be detected promptly but also because of the impact that the control of associated co-morbidities could have on the probability of in-hospital death. The Bayesian Network that we have presented allows us answer to this question.
